# Conditional expression of retrovirally delivered anti-MYCN shRNA as an in vitro model system to study neuronal differentiation in MYCN-amplified neuroblastoma

**DOI:** 10.1186/1471-213X-11-1

**Published:** 2011-01-03

**Authors:** Jørn R Henriksen, Bjørn Helge Haug, Jochen Buechner, Ellen Tømte, Cecilie Løkke, Trond Flaegstad, Christer Einvik

**Affiliations:** 1 Department of Pediatrics, University Hospital of North-Norway, 9038 Tromsø, Norway; 2 Department of Pediatric Research, Institute of Clinical Medicine, University of Tromsø, 9037 Tromsø, Norway

## Abstract

**Background:**

Neuroblastoma is a childhood cancer derived from immature cells of the sympathetic nervous system. The disease is clinically heterogeneous, ranging from neuronal differentiated benign ganglioneuromas to aggressive metastatic tumours with poor prognosis. Amplification of the MYCN oncogene is a well established poor prognostic factor found in up to 40% of high risk neuroblastomas.

Using neuroblastoma cell lines to study neuronal differentiation *in vitro *is now well established. Several protocols, including exposure to various agents and growth factors, will differentiate neuroblastoma cell lines into neuron-like cells. These cells are characterized by a neuronal morphology with long extensively branched neurites and expression of several neurospecific markers.

**Results:**

In this study we use retrovirally delivered inducible short-hairpin RNA (shRNA) modules to knock down *MYCN *expression in *MYCN*-amplified (MNA) neuroblastoma cell lines. By addition of the inducer doxycycline, we show that the Kelly and SK-N-BE(2) neuroblastoma cell lines efficiently differentiate into neuron-like cells with an extensive network of neurites. These cells are further characterized by increased expression of the neuronal differentiation markers *NFL *and *GAP43*. In addition, we show that induced expression of retrovirally delivered anti-*MYCN *shRNA inhibits cell proliferation by increasing the fraction of MNA neuroblastoma cells in the G1 phase of the cell cycle and that the clonogenic growth potential of these cells was also dramatically reduced.

**Conclusion:**

We have developed an efficient *MYCN*-knockdown *in vitro *model system to study neuronal differentiation in MNA neuroblastomas.

## Background

Neuroblastoma is a childhood cancer arising from the sympaticoadrenal lineage of the neural crest. It is characterized by diverse clinical behaviours ranging from spontaneous regression, maturation to more benign forms (ganglioneuroblastoma or ganglioneuroma), to rapid tumour progression and death [1]. Amplification of the *MYCN *oncogene has been considered the most important prognostic factor for progressive disease and poor outcome. Despite intense efforts to elucidate a mechanism by which *MYCN *overexpression acts to promote the aggressive phenotype, the functional roles of the MYCN protein in neuroblastoma are poorly understood [2].

Several alternative mechanisms for neuroblastoma regression have been proposed over the past 20 years, although the principal mechanism underlying this peculiar phenomenon remains to be fully elucidated [3-5]. Tumour maturation via neuronal differentiation has recently been proposed as a plausible candidate mechanism to explain neuroblastoma regression [6]. Therefore, the study of neuroblastoma as a model system for the general process of tumour cell differentiation, as well as neuronal development, is important to reveal the secrets of both tumour maturation and spontaneous regression.

Using neuroblastoma cell lines to study neuronal differentiation *in vitro *is now well established. Induced neuron-like morphological and biochemical changes to the SH-SY-5Y neuroblastoma cell line was demonstrated almost 30 years ago using a bioactive phorbolester as the inducing agent [7]. Since then, a variety of various agents and growth factors have been shown to induce neuronal differentiation in many neuroblastoma cell lines (reviewed in [8]). Furthermore, specific suppression of *MYCN *expression using traditional antisense techniques or small interfering RNA molecules (siRNA) have also been shown to promote neuronal differentiation in several *MYCN*-amplified (MNA) neuroblastoma cell lines [9-12].

Plasmid and viral vector-based systems containing RNA polymerase III promoters for the expression of short hairpin RNAs (shRNAs) have become useful tools for modulating gene expression in mammalian cells [13]. Compared to siRNAs, the use of shRNAs to suppress gene function has been demonstrated to be more effective [14]. In addition, shRNA-based strategies offer the advantage of inducible expression in situations in which gene knockdown is expected to have a deleterious effect on the targeted cell.

Retroviral expression systems have proven to be useful tools for sustained long-term expression of transgenes in mammalian cells [15]. With the development of protocols to produce high-titer infectious, replication-incompetent retroviral particles, these expression systems are now commonly used for shRNA delivery.

In this study, we have developed a retroviral tetracycline-inducible anti-*MYCN *shRNA expression system to study MYCN knockdown-mediated neuronal differentiation in MNA neuroblastoma cell lines. We reveal that MNA neuroblastoma cell lines induced to express the anti-*MYCN *shRNAs efficiently undergo morphological and biochemical changes consistent with neuronal differentiation.

## Results and Discussion

### Retrovirally delivered inducible shRNA expression in HEK293T-Rex cells

We have previously developed and characterized an efficient doxycyclin (dox)-inducible variant of the H1 promoter (H1-2O2-US/DS) for shRNA expression in human cells. The H1-2O2-US/DS promoter was shown to be almost completely inactive in the non-induced state, while induction by dox yielded a high shRNA expression in transient transfection studies using an anti-luciferase (anti-luc) shRNA reporter system [16].

**Figure 1:**
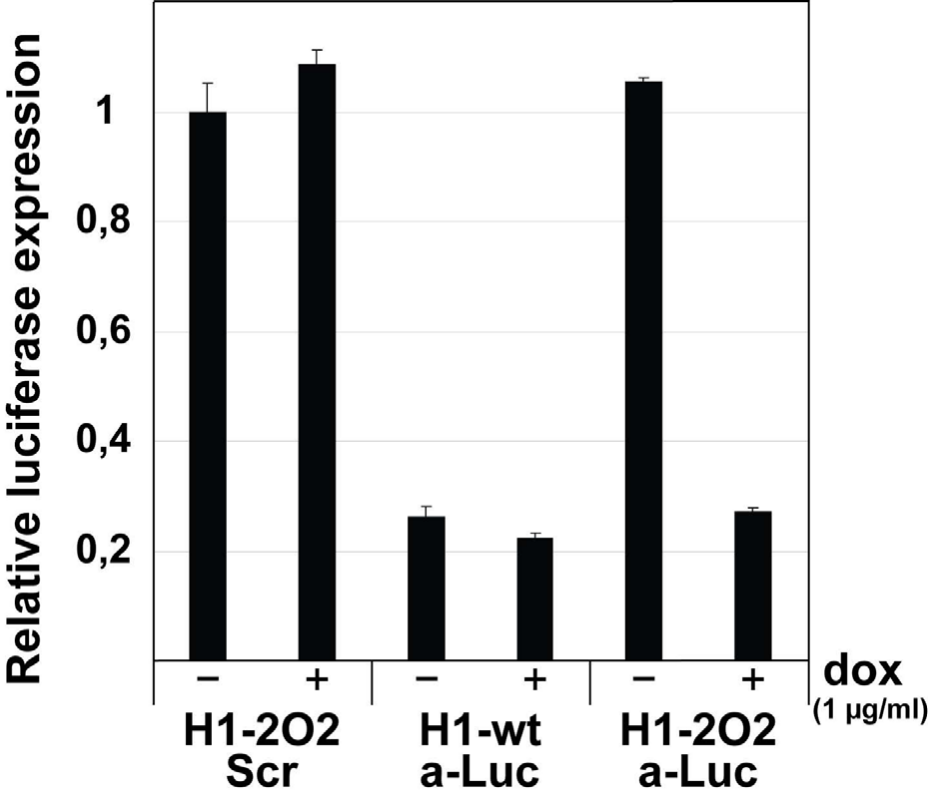
**Retrovirally delivered inducible shRNA expression in HEK293 cells**. TetR-expressing HEK293 cells (HEK293T-Rex) were transduced with retroviruses expressing anti-Luciferase (a-Luc) or scrambled (Scr) shRNAs. The a-Luc shRNA was expressed from a wt-H1 or the inducible H1-2O2 promoter in the absence (-) or presence (+) of 1 μg/ml doxycyclin (dox) for 48 hrs. Error bars indicate SDs.

As a first step towards developing a long-term inducible shRNA expression system, we subcloned the previously described anti-luc shRNA constructs and the corresponding scrambled control shRNA into a retroviral expression vector. shRNA-expressing retroviruses were transduced into HEK293T-Rex cells followed by transient transfection of the luciferase reporter vector. Two days after transfection, cells were monitored for dox-regulated shRNA expression. Addition of dox to the transduced cells had only minor effects on luciferase expression as can be seen from both the inducible scrambled negative control (H1-2O2 Scr) and the H1-wt expressed anti-luc shRNA (H1-wt a-Luc) (Figure 1). Induction of anti-luc shRNA expression from the H1-2O2 promoter by the addition of dox downregulated the luciferase reporter to levels similar to that observed for the H1-wt promoter. At the same time, no transcriptional activity was observed in the non-induced state (H1-2O2 aLuc, -dox) when compared to the scrambled shRNA control.

These data clearly show that retroviral delivery of the H1-2O2 US/DS-inducible shRNA expression system to HEK293T-Rex cells allows an efficient inducible expression of mature shRNA molecules.

### Efficient downregulation of *MYCN *expression in MNA neuroblastoma cell lines

We have previously designed an anti-*MYCN *shRNA construct (aMN-887) for downregulation of *MYCN *oncogene expression in *MYCN*-amplified (MNA) neuroblastoma cells [17]. This design procedure was based on the original Hannon protocol for shRNA construction, recommending 29 bp stem structures [18]. We have now constructed a new anti-*MYCN *shRNA construct (aMN-1658) targeting the 3'UTR of the *MYCN *mRNA.

**Figure 2:**
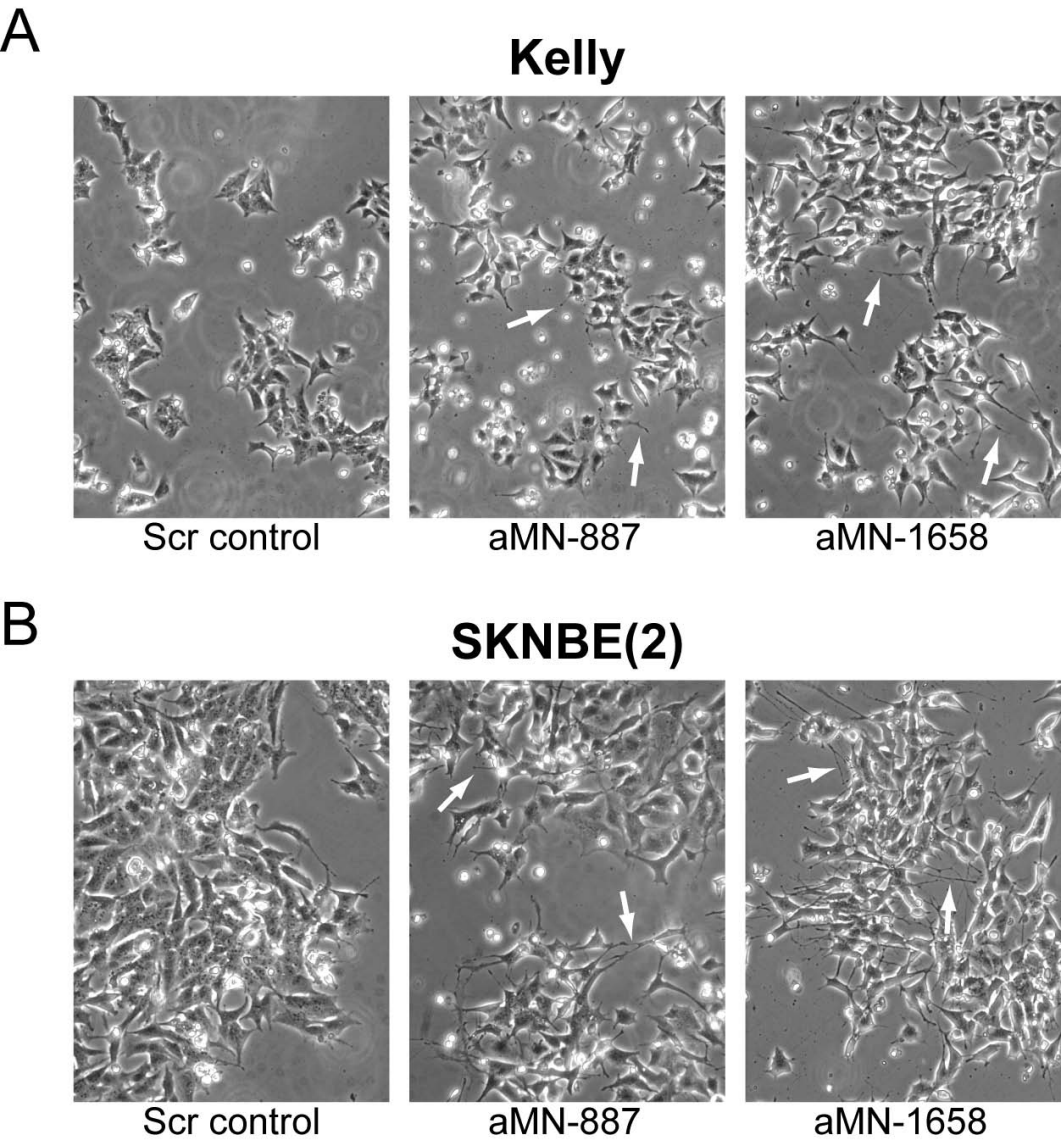
**MYCN knockdown induces neurite outgrowth in MNA neuroblastoma cell lines**. MNA Kelly **(A) **and SK-N-BE(2) **(B) **cells transiently transfected for 3 days with anti-*MYCN *(aMN-887 and aMN-1658) and scrambled (Scr control) shRNA expressing plasmids. Cells expressing the aMN shRNAs show neurite outgrowth characteristic of neuronal differentiated cells. White arrows indicate neurite outgrowth.

**Figure 3:**
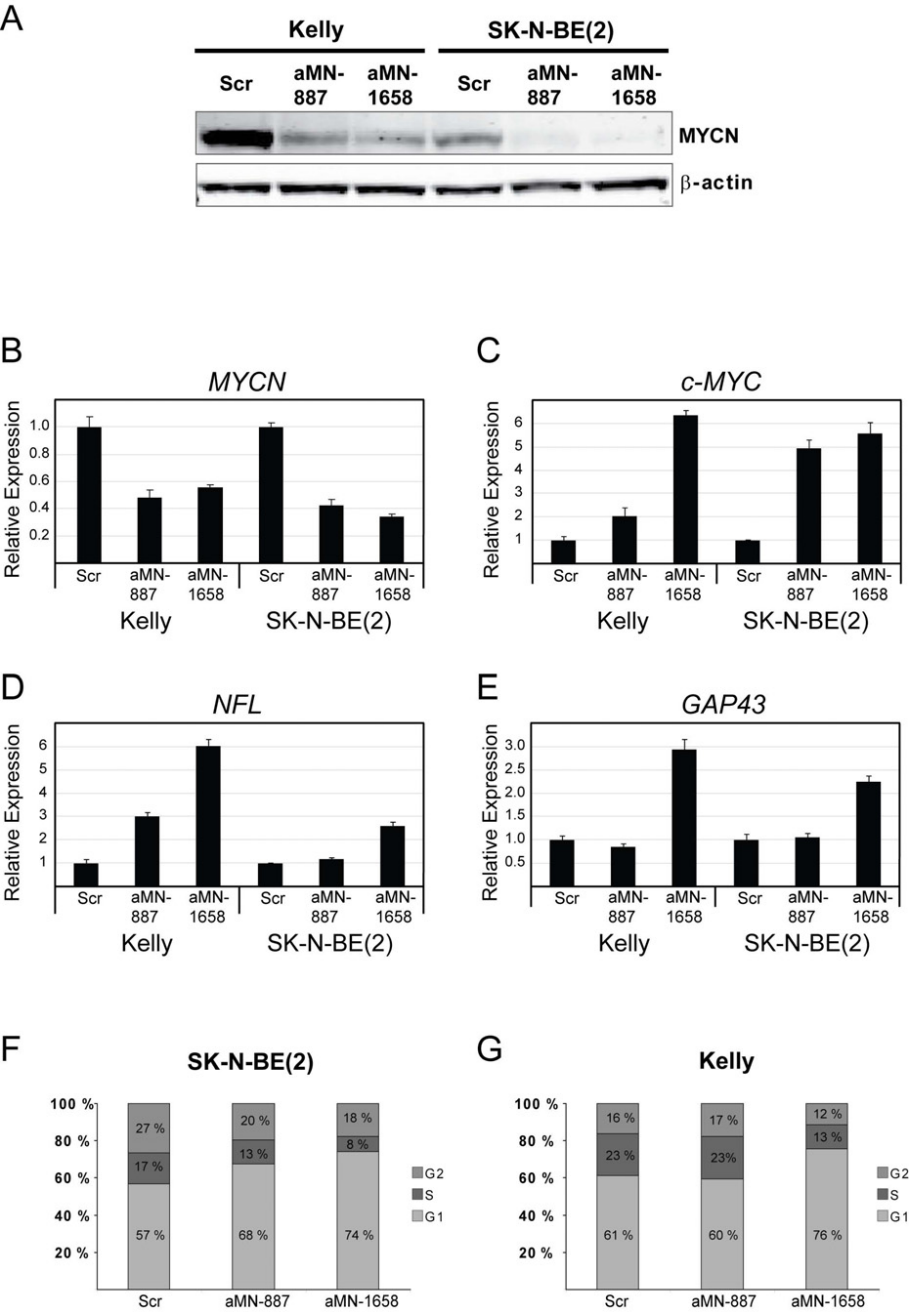
**Transient *MYCN *knockdown in MNA Kelly and SK-N-BE(2) cells using shRNA technology**. (**A): **A representative western blot analysis of MYCN protein expression in Kelly and SK-N-BE(2) cells transiently transfected for 3 days with plasmids expressing the aMN-887 and aMN1658 shRNAs. β-actin expression was used for normalization. Quantitative real-time RT-PCR was used to investigate the expression of *MYCN ***(B)**, *c-MYC ***(C)**, neurofilament light-chain - *NFL ***(D) **and growth-associated protein 43 - *GAP43 ***(E) **mRNAs. Error bars indicate SDs. (**F) **and (**G)**: Flow cytometric analyses showing the cell cycle distribution of SK-N-BE(2) and Kelly cells transiently transfected with the aMN-887 and aMN-1658 shRNA expressing plasmids. Scr indicates the scrambled shRNA controls.

The MNA Kelly and SK-N-BE(2) neuroblastoma cell lines were transiently transfected with plasmids expressing the aMN-887 or aMN-1658 shRNAs. A scrambled shRNA (Scr) construct was used as the control. Three days after transfection, cells receiving the aMN (anti-*MYCN*) shRNAs appeared to have a more neuron-like phenotype characterized by neurite outgrowth, while cells transfected with the control shRNA did not reveal any morphological alterations as compared to non-transfected cells (Figure 2). Western immunoblot and quantitative real-time RT-PCR analysis confirmed specific suppression of the MYCN protein and *MYCN *mRNA in both cell lines, respectively (Figure 3a and Figure 3b).

Consistent with previous data, an inverse correlation between *MYCN *and *c-MYC *mRNA expression was also confirmed (Figure 3c) [19,20]. The observed morphological changes were documented biochemically by increased expression of the early neuronal differentiation marker *NFL *(Figure 3d). Furthermore, expression of aMN-1658, but not of aMN-887, resulted in a marked increase in the late neuronal differentiation marker *GAP43*, which is known to be involved in axonal outgrowth and synapse formation (Figure 3e) [21]. This observation is consistent with the more extensive neuronal differentiation observed for both Kelly and SK-N-BE(2) cells transfected with aMN-1658 compared to cells transfected with aMN-887 (Figure 2).

These data show that aMN-1658 is an efficient anti-*MYCN *shRNA construct which suppresses *MYCN *expression and induces prominent neuronal differentiation in MNA neuroblastoma cell lines. For that reason, the aMN-1658 shRNA was used in the following research to knock down MYCN expression.

Previous reports document conflicting results on cell cycle distribution data of SK-N-BE(2) cells treated with anti-*MYCN *siRNAs. While Yu et al. reported no apparent difference in the fraction of G1 cells after siRNA treatment, Bell et al. showed that *MYCN *siRNA treatment increased the G1 population by 8.1% compared to a negative scrambled control siRNA [9,22]. In order to investigate the effect of shRNA-mediated *MYCN *knockdown on the cell cycle distribution pattern, we transiently transfected SK-N-BE(2) cells with plasmids expressing the aMN-887, aMN-1658 or a scrambled shRNA from a wt H1 promoter. Three days posttransfection the cell cycle distribution pattern was monitored using flow cytometry. The fraction of cells in the G1 phase of the cell cycle increased from 57% (Scr) to 74% for SK-N-BE(2) cells transfected with the aMN-1658 shRNA (Figure 3f). Similar results were obtained with aMN1658-transfected Kelly cells (Figure 3g).

A discrepancy between the knockdown levels for the MYCN protein (85-94%) and mRNA (45-70%) was observed in our *MYCN *shRNA knockdown experiments, with similar results being previously reported for *MYCN *siRNA knockdown by others [23]. In order to further document that only the expression of the *MYCN *shRNAs results in reduced MYCN expression, we set up additional experiments. The complete *MYCN *3'UTR sequence, including the aMN-1658 target sequence, was cloned behind a luciferase reporter gene in the pMIR-REPORT vector (Ambion). Co-transfecting this reporter vector and the aMN-shRNA expressing plasmids into HEK-293 cells demonstrated the specific knockdown, monitored as an 80% reduction in luciferase activity, by the aMN-1658 shRNA (Additional file 1a). When aMN-1658 was co-transfected with a control reporter vector containing a 3'UTR lacking the aMN-1658 target sequence (Luc NT-3'UTR), no change in luciferase expression was observed, and similar results were obtained in the non-MNA neuroblastoma cell line SH-SY-5Y (data not shown).

In addition, we co-expressed the aMN-shRNA constructs and the *MYCN *cDNA lacking the 3'UTR sequence in a MNA neuroblastoma cell line. Downregulation of both the endo- and exogenously expressed MYCN protein was only observed for the aMN-887 shRNA targeting the coding region of *MYCN*. The aMN-1658 shRNA was not able to suppress the exogenously expressed *MYCN *cDNA lacking the 3'UTR structure (Additional file 1b).

These experiments show that the aMN-1658 shRNA specifically reduces expression of mRNAs containing the *MYCN *3'UTR target. However, we have not been able to confirm or rule out the possibility that a part of the MYCN protein reduction by aMN-1658 shRNA knockdown is mediated by a miRNA-like mechanism not involving mRNA degradation. Nonspecific cellular effects induced by shRNA expression were excluded by showing that the expression level of several genes involved in the interferon response (*OAS2*, *MX1*, *IFITM1 *and *ISGF3γ*) remained unaltered during transfection of various shRNA constructs (data not shown).

### Retrovirally delivered inducible anti-*MYCN *shRNA expression in MNA neuroblastoma cell lines

In order to generate a long-term inducible anti-*MYCN *shRNA expression system, we first cloned the aMN-1658 shRNA module behind the inducible H1-2O2-US/DS promoter and then gated the resulting construct into a retroviral expression vector to generate pRV-1658. A similar vector expressing a scrambled shRNA was constructed as a negative control (pRV-Scr).

**Figure 4:**
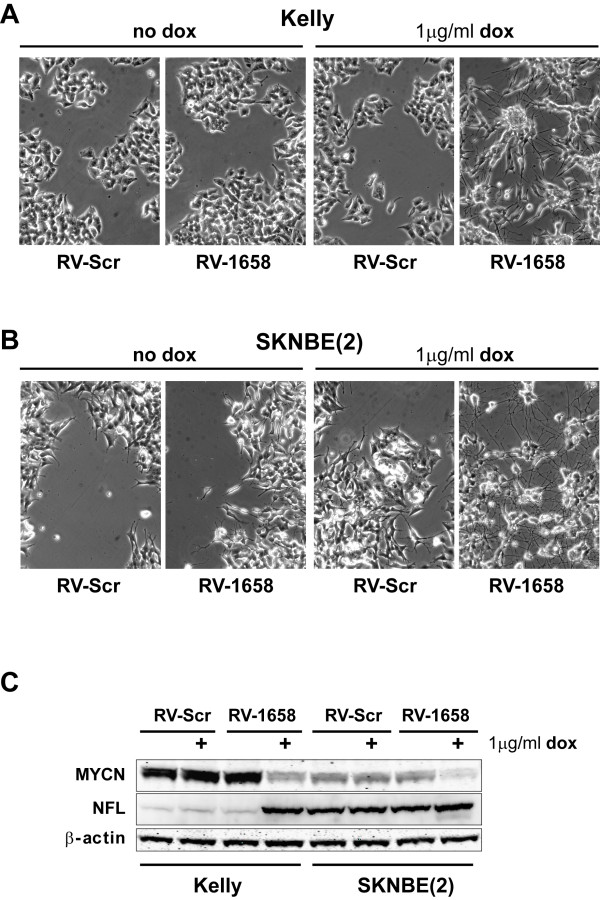
**Inducible *MYCN *knockdown in retrovirally transduced MNA neuroblastoma cell lines**. Representative micrographs showing the morphology of Kelly **(A) **and SK-N-BE(2) **(B) **cells transduced with retroviruses delivering the dox-inducible aMN-1658 shRNA module (RV-1658). RV-Scr indicates the cells receiving retroviruses delivering an inducible scrambled shRNA module. **(C): **A representative western blot showing MYCN and NFL expression in Kelly and SK-N-BE(2) cells transduced with retrovirus RV-1658 and induced to express the shRNA by addition of 1 μg/ml doxycyclin (dox).

**Figure 5:**
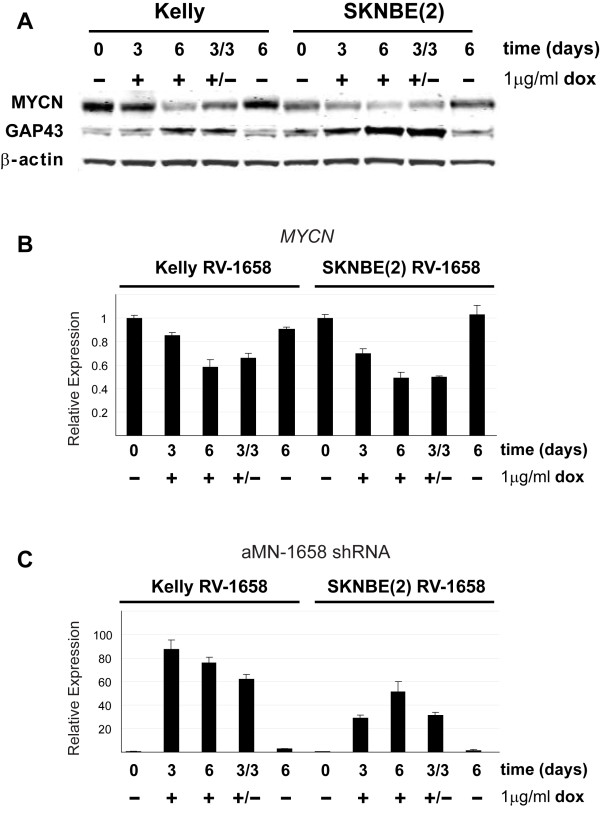
**Induced anti-MYCN shRNA expression decreases MYCN and increases GAP43 expression in MNA neuroblastoma cells**. **(A) **A representative western blot analysis of MYCN, GAP43 and β-actin protein expression in Kelly and SK-N-BE(2) cells induced to express the aMN-1658 shRNA. Real-time RT-PCR analysis of *MYCN *mRNA **(B) **and aMN-1658 shRNA **(C) **expression was performed on total RNA isolated from Kelly and SK-N-BE(2) cells treated as described. Cells were transduced with retrovirus RV-1658 and incubated for the indicated numbers of days in the presence (+) or absence (-) of 1 μg/ml doxycyclin (dox). 3/3 and +/- indicate that the cells were incubated for 3 days in the presence of dox, followed by 3 days in the absence of dox. Error bars indicate SDs.

Kelly and SK-N-BE(2) neuroblastoma cell lines stably expressing the TetR (tetracycline repressor) were transduced with the retroviruses RV-1658 and RV-Scr and then incubated for 6 days in a growth medium both with and without dox. As can be seen from Figure 4a and Figure 4b, only the cells induced to express the aMN-1658 shRNA demonstrated prominent neuronal differentiation. The lack of morphological changes in non-induced RV-1658 transduced cells indicates that the H1-2O2 promoter shows minimal transcriptional leakage in the absence of dox. The addition of dox to RV-Scr transduced cells had no significant effect on the cell morphology. Western blot analysis of the retrovirally transduced Kelly and SK-N-BE(2) cells revealed efficient repression of MYCN expression in cells induced to express the aMN-1658 shRNA (Figure 4c). Similar to the observations made in transient expression analyses, the *MYCN *mRNA levels were reduced to a lesser extent when compared to the western blot data (data not shown and Figure 5b).

In order to investigate the reversibility of the inducible retroviral shRNA expression system, we performed a time-course experiment in which the aMN-1658 expression was turned on for 3 days before removing dox from the media for another 3 days to eliminate shRNA expression.

Compared to the differentiated cells continuously exposed to aMN-1658 shRNA for 6 days, no significant change in cell morphology was observed when shRNA expression was turned off (data not shown). When the MYCN protein and mRNA levels were measured during the 6 days of shRNA induction, a steady decrease in MYCN expression was observed. Removal of dox from the media after 3 days of exposure did not efficiently recover MYCN expression. Consistent with the observed neuron-like phenotype, GAP43 protein levels remained high after dox removal (Figure 5a and Figure 5b).

By the use of a quantitative RT-PCR protocol designed to measure the mature antisense shRNA strand, we observed a maximum 90-fold increase in aMN-1658 RNA after 3 days of dox induction in the Kelly cells (Figure 5c). After additional 3 days of induction, a slight decrease in shRNA expression was observed. For the SK-N-BE(2) cell line, a maximum 50-fold increase in shRNA expression was observed after 6 days of continuous induction. Removing the inducing agent from the media did not efficiently cease shRNA expression. We suggest that this lack of reversibility is most likely due to high intracellular shRNA stability and/or insufficient removal of dox from the media.

We also analysed the expression of the neuronal differentiation markers *NFL *and *GAP43 *in Kelly and SK-N-BE(2) cells transduced with shRNA-expressing retroviruses. Consistent with the observed morphological changes towards a neuronal phenotype, both differentiation markers increased upon aMN-1658 induction (Figure 5a, Additional file 2a and Additional file 2b). Quantitative real-time PCR revealed a 5-8 fold increase in *NFL *mRNA and a 3-fold increase in *GAP43 *mRNA in both cell lines induced to express the aMN-1658 shRNA.

SK-N-BE(2) has previously been described as a noradrenergic neuroblastoma cell line based on expression of enzymes involved in neurotransmitter synthesis [24]. In order to get further information about the neuronal phenotype observed in our experiments, we investigated the expression levels of the cholinergic markers choline acetyl transferase (*ChAT*) and vesicular acetylcholine transporter (*VAChT*), and the catecholaminergic marker tyrosine hydroxylase (*TH*). When Kelly and SK-N-BE(2) cells were induced to differentiate by *MYCN *knockdown we observed increased mRNA expression levels of *ChAT *and *VAChT*. The mRNA levels of *TH *decreased during the same treatment (Additional file 2c). These data are consistent with a switch towards a more sympathetic cholinergic neuronal phenotype. A similar switch in neuronal phenotype, including increased *NPY *expression, has previously been reported for a neuroblastoma cell line induced to differentiate by a combination of retinoic acid (RA) and brain-derived neurotropic factor (BDNF) [25]. Differentiated neuroblastoma tumours have recently been shown to have cholinergic characteristics as compared to poorly-differentiated or undifferentiated neuroblastomas [26].

In summary, our data show that retroviral delivery of inducible anti-*MYCN *shRNAs to MNA neuroblastoma cells efficiently reduces MYCN protein expression and induces neuronal differentiation.

### Induced anti-*MYCN *shRNA expression inhibits cell proliferation and clonogenic growth of MNA neuroblastoma cells

**Figure 6:**
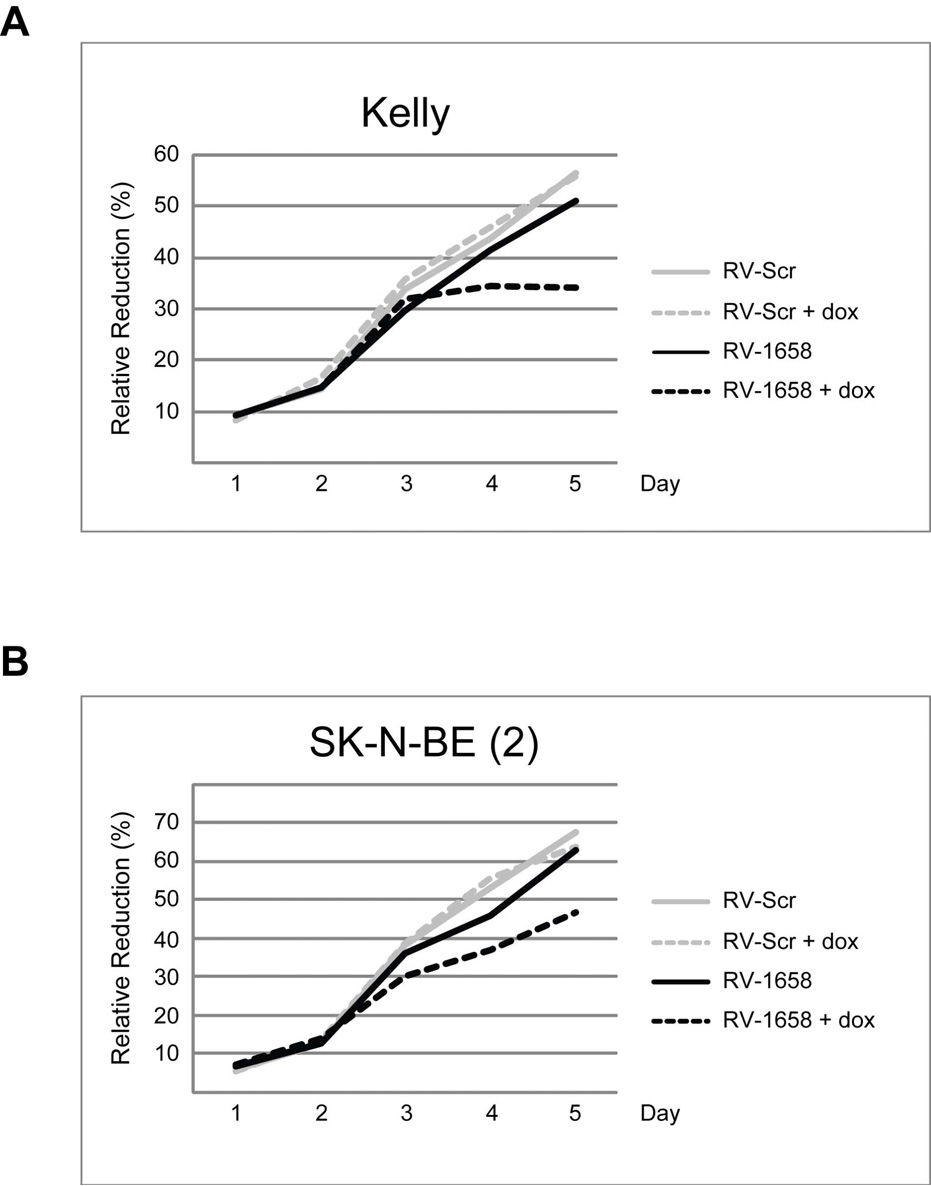
**The proliferative inhibition effect of induced *MYCN *knockdown in MNA neuroblastoma cells**. The Alamar Blue Assay was used to measure cell viability in Kelly **(A) **and SK-N-BE(2) **(B) **cells transduced with retrovirus RV-Scr (scrambled control) and RV-1658 (anti-*MYCN*). The addition of doxycyclin (+ dox) induces expression of the shRNAs. AB reduction = Alamar Blue reduction.

The Alamar Blue Assay was used to measure cell proliferation in MNA neuroblastoma cells induced to express the anti-*MYCN *shRNA from retroviral vectors. Figure 6 shows that the rate of cell proliferation was selectively decreased from day 2 in SK-N-BE(2) and day 3 in Kelly cells induced to express the aMN-1658 shRNA. Cells expressing the scrambled shRNA showed no differences in cell proliferation due to the presence or absence of dox. The slight reduction in cell proliferation observed in the non-induced aMN-1658 transduced cells is most likely due to a small leakage from the shRNA-expressing promoter. The delayed decrease in cell proliferation observed in Kelly cells is consistent with the ~5-fold higher *MYCN *mRNA levels in comparison to MNA SK-N-BE(2) [27,28].

The observed reduction in the proliferation of cells induced to express the anti-*MYCN *shRNA was further investigated by flow cytometry in order to elucidate the cell cycle distribution pattern. Induced *MYCN *knockdown in the MNA Kelly cells increased the fraction of G1 cells from 63% to 72%. For the MNA SK-N-BE(2) cells the fraction of G1 cells increased from 46% to 57% upon induction of aMN-1658 expression. Additon of dox alone had no effect on the cell cycle distribution pattern in cells transduced with retroviruses expressing a scrambled shRNA control (Additional file 3).

**Figure 7:**
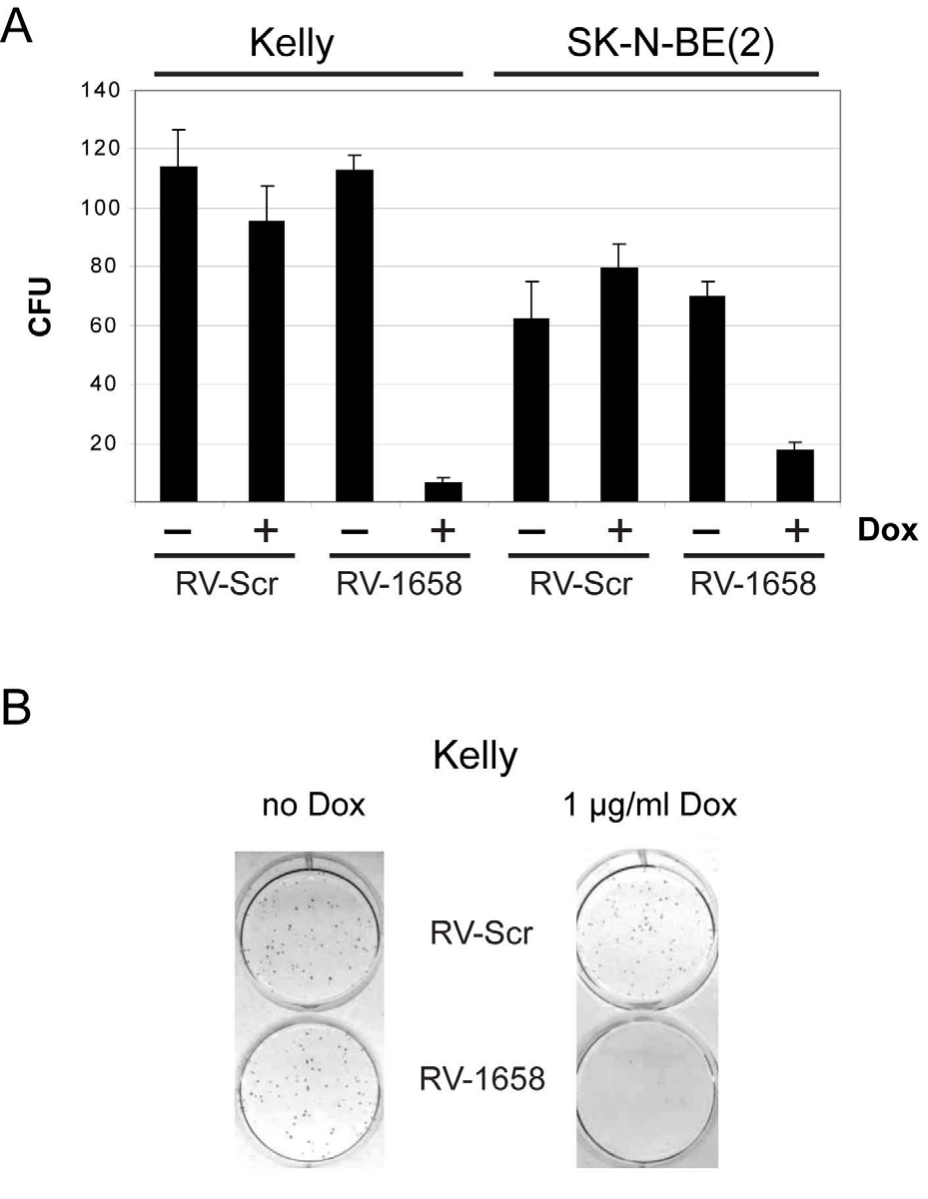
**Reproductive cell survival in MNA neuroblastoma cells after *MYCN *knockdown**. **(A)**: Graphic presentation of colony forming units (CFU) after induced expression (+ dox) of scrambled (Scr) and anti-*MYCN *(aMN1658) shRNA in Kelly and SK-N-BE(2) cells. **(B)**: Representative pictures of CFU from retrovirally transduced Kelly cells as presented in A.

Finally, *in vitro *clonogenic assays were used to measure the reproductive cell survival in MNA neuroblastoma cells induced to differentiate by *MYCN *knockdown. Figure 7 shows that *MYCN *knockdown leads to dramatic growth inhibition only in MNA neuroblastoma cells induced to express the anti-*MYCN *shRNA.

Together, these data indicate that the induced expression of retrovirally delivered anti-*MYCN *shRNA inhibits cell proliferation by increasing the fraction of MNA neuroblastoma cells in the G1 phase of the cell cycle. The clonogenic growth potential of these cells was also dramatically reduced.

## Conclusion

We have developed an efficient *MYCN*-knockdown *in vitro *model system to study neuronal differentiation in MNA neuroblastomas.

## Methods

### Cell cultures and transfection

The human *MYCN*-amplified neuroblastoma cell lines Kelly and SK-N-BE(2), and their derivatives SKNBE(2)-TetR and Kelly-TetR (constitutively expressing the Tetracycline Repressor), were cultivated as previously described [16]. HEK293 and Hek293 Phoenix-amphopack (a kind gift from Dr. James Lorens) cells were grown in a Dulbecco's modified Eagle's medium (DMEM) [29]. HEK293-TREx cells (Invitrogen, Carlsbad, CA, USA) were grown in DMEM with 15 μg/ml blasticidin. All media were supplemented with 10% FBS. Cells were maintained in a humidified 37°C incubator with 5% CO_2_, supplied with a fresh complete medium every 3 days and subcultured before confluence was reached.

Cell lines were transfected using Lipofectamine 2000 (Invitrogene) according to the manufacturer's instructions.

### Molecular cloning

The number denotation of anti-*MYCN *(aMN) shRNAs describes the first position of the shRNA target recognition site in the *MYCN *cDNA sequence (NM_005378).

Construction of the following shRNAs was performed by annealing a sense and an antisense oligonucleotide: aMN-887 (ON106/ON107) and aMN-1658 (ON413/ON414). Oligonucleotide (ON) sequences are listed in Additional file 4. Annealed oligonucleotides were ligated into BglII/HindIII digested pENTR-H1-wt or pENTR-H1-2O2-US/DS vectors as previously described [16] to generate paMN-887/H1wt, paMN-887/H1-2O2, paMN-1658/H1wt and paMN-1658/H1-2O2. Scrambled (Scr) shRNA expressing vectors pScr/H1wt and pScr/H1-2O2 were made in a similar way using ON110/ON111. The vectors paMN-887/H1wt and paMN-1658/H1wt were used in transient transfection studies.

To generate the inducible retroviral expression vectors pRV-1658 and pRV-Scr, the shRNA expression modules from paMN-1658/H1-2O2 and pScr/H1-2O2 respectively, were gated into the retroviral destination vector L193 RRI-GreenattR1ccdBCmRattR2 (a kind gift from Dr. David Micklem) using the Gateway LR Clonase Enzyme Mix (Invitrogen). L193 RRI-GreenattR1ccdBCmRattR2 is based on the L071 RRI-Green vector [30]. The U6promoter/shRNA cloning cassette of L071 (flanked by HindIII and SalI sites) was replaced with an attR1-ccdB-CmR-attR2 Gateway cloning cassette derived from pDONR221 (Invitrogen). This cassette allows L193 to be used as a Gateway Destination Vector and requires that the vector is propagated in a ccdB-resistant strain. All plasmid constructs were verified by DNA sequencing.

### Production of retroviruses, transduction and induction of shRNA expression

Retroviral destination vectors pRV-1658 and pRV-Scr were transfected into the Hek293-Phoenix packaging cell line seeded in 6-well plates. 24 h after transfection, the culture media were replaced by fresh media corresponding to the cell line being transduced. After another 24 h of incubation, the media were passed through a Millex HV 0.45 μm PVDF filter (Millipore, Bedford, MA, USA) to isolate retroviral particles. Polybrene (Sigma, St. Louis, MO, USA) was added to a final concentration of 4 μg/ml.

Cells were transduced by replacing the growth media with the solution of isolated retroviruses containing polybrene. The following day the virus-containing media were replaced with normal growth media containing puromycin (HEK293T-Rex and Kelly: 200 ng/ml, SK-N-BE(2): 2500 ng/ml).

Induction of shRNA expression was performed by the addition of 1 μg/ml doxycyclin (dox) to the media.

### Quantitative real-time RT-PCR

Total RNA was extracted using the Qiagen miRNeasy Mini Kit (Qiagen, Hilden, Germany). 1 μg total RNA was reverse transcribed in 20 μl using a Qiagen miScript Reverse Transcription Kit according to the manufacturer's instructions. Real-time PCR analysis was performed in 25 μl reactions using *Power *SYBR Green PCR Master Mix (Applied Biosystems, Warrington, UK) as recommended by the manufacturer (2.5 μl of a 20×-diluted RT-reaction as template, 0.2 μM of each primer). *UBC *(ON56/ON57) and *PPIA *(ON174/ON175) were used as reference genes for normalizing expression levels of *MYCN *(ON440/ON441 and ON145/146 were used for comparing the effect of aMN-887 and aMN-1658), *NFL *(ON58/ON59), *GAP43 *(ON298/ON299), *c-myc *(ON100/ON101), TH (ON453/ON454), ChAT (ON516/ON517), VAChT (ON518/ON519) and NPY (ON060/ON061). Primer sequences are listed in Additional file 4.

Quantification of the aMN-1658 shRNA was performed using miScript SYBR green PCR Kit (Qiagen) in 50 μl reactions with 1 μl of the 20×-diluted RT-reaction as template. Specific primers for quantifying aMN-1658 shRNA were ordered as a custom miScript Primer Assay from Qiagen using 5'CACACAAGGUGACUUCAACAGUU3' as the mature miRNA sequence, and were used at concentrations specified by the manufacturer. *UBC *and *PPIA *were used for normalizing. All quantitative PCR reactions were performed using the Applied Biosystems 7300 Real-Time PCR System (Applied Biosystems) with thermal profiles as recommended by the manufacturer. The fold change in mRNA and miRNA levels were calculated using the ΔΔCT method with the qBASE software [31].

### Western blotting

Cells were trypsinized and lysed in a Tropix Lysis Solution (Bedford, MA, USA). Lysate cleared by centrifugation was measured for total protein using Bio-Rad *DC *protein assay (Bio-Rad, Hercules, CA, USA), and 35 μg protein were loaded in each well on pre-casted NuPAGE 4-12% Bis-Tris Gels (Invitrogen). The separated proteins were transferred to an Immobilon-FL PVDF transfer membrane (Millipore) and blocked for 1 hour at room temperature in Odyssey Blocking Buffer (LI-COR, Lincoln, NE, USA) before incubation at 4°C overnight with the following primary antibodies: mouse anti-NMYC (Calbiochem/Merck, Darmstadt, Germany), mouse anti-GAP43 (Abcam, Cambridge, UK), goat anti-NFL (Santa Cruz Biotech., Santa Cruz, CA, USA) and rabbit anti-βActin (Sigma) diluted in the blocking buffer. Secondary antibodies were goat anti-rabbit IRDye800CW (Rockland, Gilbertsville, PA, USA) and goat anti-mouse Alexa Fluor 680 (Invitrogen). Antibody binding was detected using the Odyssey Infrared Imaging System (LI-COR). NFL was detected by HRP conjugated rabbit anti-goat secondary antibody (DAKO, Glostrup, Denmark) and SuperSignal West Pico Chemiluminescent Substrate (Pierce, Rockford, IL, USA).

### Luciferase assay

Retrovirus-transduced HEK293T-Rex cells were seeded in 12-well plates. 48 hrs following induction with 1 μg/ml dox, each well was transfected with 0.02 μg pGL4.75[hRluc/CMV] Vector (Promega, Madison, WI, USA) and 0.1 μg pGL3 Control Vector (Promega). Firefly and renilla luciferace activity were measured after 48 hrs using the Dual-Luciferase Reporter Assay System (Promega) according to the manufacturer's instructions. Luminescence was measured on a Luminoskan Ascent luminometer (Thermo Sci., Waltham, MA, USA). Renilla luciferase was used to normalize the data, and all experiments were performed in triplicate.

### Clonogenic assay

The cells were grown for 7-11 days with replacement of the media every third day. On the last day of the experiment, the cells were washed once in 1 × PBS and stained with the clonogenic reagent (50% EtOH, 0.25% 1,9-dimethyl-methylene blue) for 45 min. Cells were washed twice in PBS before visible colonies were counted.

### Cell viability assay

The cytotoxic effect of induced *MYCN *knockdown on MNA neuroblastoma cells was analysed using the Alamar Blue Assay according to the manufacturer's instructions. In brief, the cells were seeded at a density of 40000 (Kelly) and 15000 (SK-N-BE(2)) cells per well in 12-well plates. Media were replaced every second day. On the indicated days, 100 μl of Alamar Blue solution were added to each well and incubated for 4 hrs at 37°C. Absorbance at 570 and 600 nm was measured on a plate reader and the relative reduction of Alamar Blue was calculated as described by the manufacturer. The calculated average relative reduction from three independent experiments was calculated.

### Flow cytometric analysis of cell cycle distribution

Cells were harvested using Trypsin-EDTA (Sigma-Aldrich) and washed once in 1 × PBS. The cells were then fixed for 2 hrs in ice-cold 70% EtOH. After fixation, the EtOH was removed by centrifugation and the cells were washed once in 1 × PBS before being stained for 30 min at room temperature in a propidium iodide (PI)-staining solution (PBS with 20 μg/ml PI (Sigma), 60 μg/ml RNase A (Sigma) and 0.1% v/v Triton X-100 (Sigma)). Fluorescence emitted from the PI-DNA complex was analysed by flow cytometry, using a FACS Aria Flow Cytometer (BD Biosciences, San Jose, CA, USA).

## Competing interests

The authors declare that they have no competing interests.

## Authors' contributions

JRH designed and constructed the vectors, performed most of the experiments, drafted the manuscript, and critically planned and discussed all aspects of this work. BHH performed the clonogenic assays, participated in transduction studies. BHH, JB and TF helped to plan and critically discuss the study and participated in drafting the manuscript. ET performed several real-time RT-PCR experiments and cultivated the retrovirus transduced neuroblastoma cells. CL cultivated the cell cultures and participated in the experimental work involving cell cultures and luciferase measurements. CE designed and supervised the experimental work and wrote the final manuscript. All authors read and approved the final manuscript.

## Supplementary Material

Additional file 1 **Specific *MYCN *3'UTR knockdown mediated by the aMN-1658 shRNA**. **(A)**: The 3'UTR of *MYCN *was PCR-amplified from human genomic DNA with primers ON178 (5'AAAGCTGCGCACTAGTATCTGGACCAGGCTGTGGGTAGA3' -SpeI site) and ON181 (5' GATCAAGCTTAATTTTAAGCTATTTATTTT 3' -HindIII site). PCR products were digested with SpeI/HindIII and ligated into SpeI/HindIII-digested pMIR-REPORT vector (Invitrogen) to produce Luc-*MYCN*-3'UTR. A control Luc no-target (NT)-3'UTR plasmid was made by amplification of the 3'UTR region from REIC using ON361 (5'GATCAAGCTTAATTTTAAGCTATTTATTTT3'-SpeI site) and ON327 (5'GATCAAGCTTCTATGGAAGATTTTTAATACAGG3' -HindIII) as primers and ligated into SpeI/HindIII digested pMIR-REPORT vector using the In-Fusion Dry-Down PCR Cloning Kit (Clontech). HEK-293 cells were seeded in 12-well plates, incubated for 48 hrs and transfected with a cocktail containing: 0.02 μg pGL4.75[hRluc/CMV] Vector (Promega), 0.1 μg pMIR-Report containing either the *MYCN *3'UTR (Luc *MYCN*-3'UTR) or control (Luc-NT-3'UTR), and 1 μg shRNA expressing plasmid (pScr/H1wt, paMN-887/H1wt or paMN-1658/H1wt). Transfected cells were then incubated for 48 hrs before luciferase activities were measured as described.
**(B): **Western blot analysis of MYCN and β-actin expression in SK-N-BE(2) transfected cells. CMV-MYCN (kind gift from Dr. Jason Shohet) expresses the MYCN cDNA lacking a 3'UTR. SK-N-BE(2) cells were transiently co-transfected with the shRNA-expressing plasmids (pScr/H1wt, paMN-887/H1wt or paMN-1658/H1wt) and pCMV-MYCN (+) or the shRNA-expressing plasmids and pCMV-GFP (-). The aMN-1658 shRNA is not able to suppress expression of MYCN from the 3'UTR-lacking pCMV-MYCN plasmid. M = Magic Mark XP (Invitrogen). Click here for file

Additional file 2 **mRNA expression of neuronal markers in neuroblastoma cell lines after transduction with inducible anti-*MYCN *shRNA expressing retroviruses**. Real-time RT-PCR analysis of *NFL ***(A) **and *GAP43 ***(B) **mRNA expression in Kelly and SK-N-BE(2) cells transduced with the retrovirus RV-1658. Cells were incubated for the indicated numbers of days in the presence (+) or absence (-) of 1 μg/ml doxycyclin (dox). 3/3 and +/- indicate that the cells were incubated for 3 days in the presence of dox, followed by 3 days in the absence of dox. **(C)**: Real-time RT-PCR analysis of *ChAT, VAChT, TH *and *NPY *mRNA in RV-1658 transduced Kelly and SK-N-BE(2) cells grown in the absence (-) or presence (+) of doxycyclin (dox) for 6 days. Click here for file

Additional file 3 **Cell cycle distribution of neuroblastoma cell lines after transduction with inducible anti-*MYCN *shRNA expressing retroviruses**. Flow cytometric analysis showing the cell cycle distribution of Kelly **(A) **and SK-N-BE(2) **(B) **cells transduced with the RV-1658 and RV-Scr retroviruses in the presence (+) or absence (-) of 1 μg/ml doxycyclin (dox). Click here for file

Additional file 4 **Oligonucleotides**. Oligonucleotides used in this study. Click here for file
