# Supplementary figures and images for: Conditional expression of retrovirally delivered anti-MYCN shRNA as an in vitro model system to study neuronal differentiation in MYCN-amplified neuroblastoma
Source: BMC Dev Biol. 2011 Jan 3;11:1. doi: 10.1186/1471-213X-11-1 (PMC3022612; doi:10.1186/1471-213X-11-1)

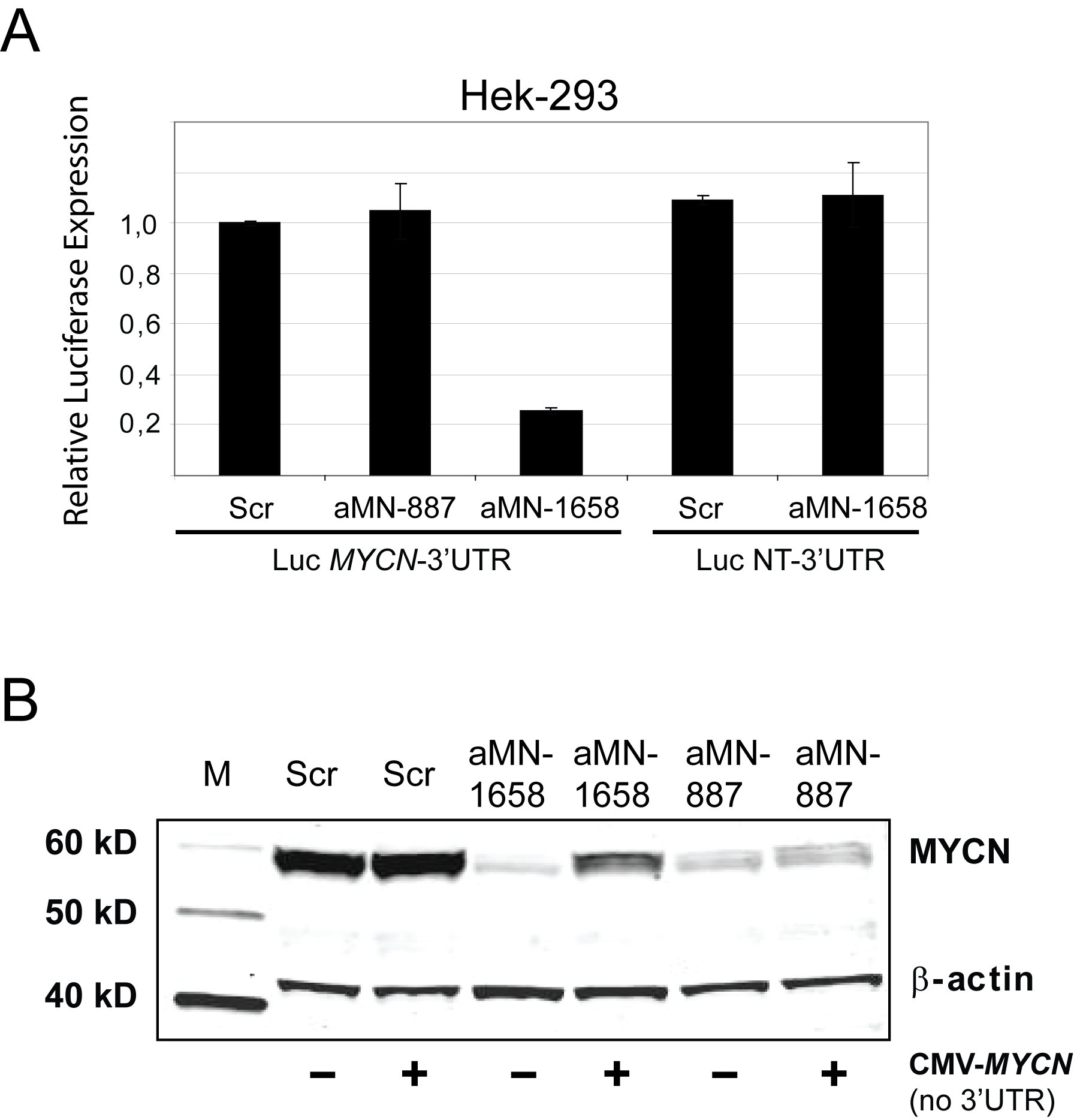

Supplement: Additional file 1 — Specific MYCN 3'UTR knockdown mediated by the aMN-1658 shRNA. (A): The 3'UTR of MYCN was PCR-amplified from human genomic DNA with primers ON178 (5'AAAGCTGCGCACTAGTATCTGGACCAGGCTGTGGGTAGA3' -SpeI site) and ON181 (5' GATCAAGCTTAATTTTAAGCTATTTATTTT 3' -HindIII site). PCR products were digested with SpeI/HindIII and ligated into SpeI/HindIII-digested pMIR-REPORT vector (Invitrogen) to produce Luc-MYCN-3'UTR. A control Luc no-target (NT)-3'UTR plasmid was made by amplification of the 3'UTR region from REIC using ON361 (5'GATCAAGCTTAATTTTAAGCTATTTATTTT3'-SpeI site) and ON327 (5'GATCAAGCTTCTATGGAAGATTTTTAATACAGG3' -HindIII) as primers and ligated into SpeI/HindIII digested pMIR-REPORT vector using the In-Fusion Dry-Down PCR Cloning Kit (Clontech). HEK-293 cells were seeded in 12-well plates, incubated for 48 hrs and transfected with a cocktail containing: 0.02 μg pGL4.75[hRluc/CMV] Vector (Promega), 0.1 μg pMIR-Report containing either the MYCN 3'UTR (Luc MYCN-3'UTR) or control (Luc-NT-3'UTR), and 1 μg shRNA expressing plasmid (pScr/H1wt, paMN-887/H1wt or paMN-1658/H1wt). Transfected cells were then incubated for 48 hrs before luciferase activities were measured as described. (B): Western blot analysis of MYCN and β-actin expression in SK-N-BE(2) transfected cells. CMV-MYCN (kind gift from Dr. Jason Shohet) expresses the MYCN cDNA lacking a 3'UTR. SK-N-BE(2) cells were transiently co-transfected with the shRNA-expressing plasmids (pScr/H1wt, paMN-887/H1wt or paMN-1658/H1wt) and pCMV-MYCN (+) or the shRNA-expressing plasmids and pCMV-GFP (-). The aMN-1658 shRNA is not able to suppress expression of MYCN from the 3'UTR-lacking pCMV-MYCN plasmid. M = Magic Mark XP (Invitrogen). [file 1471-213X-11-1-S1.PNG]

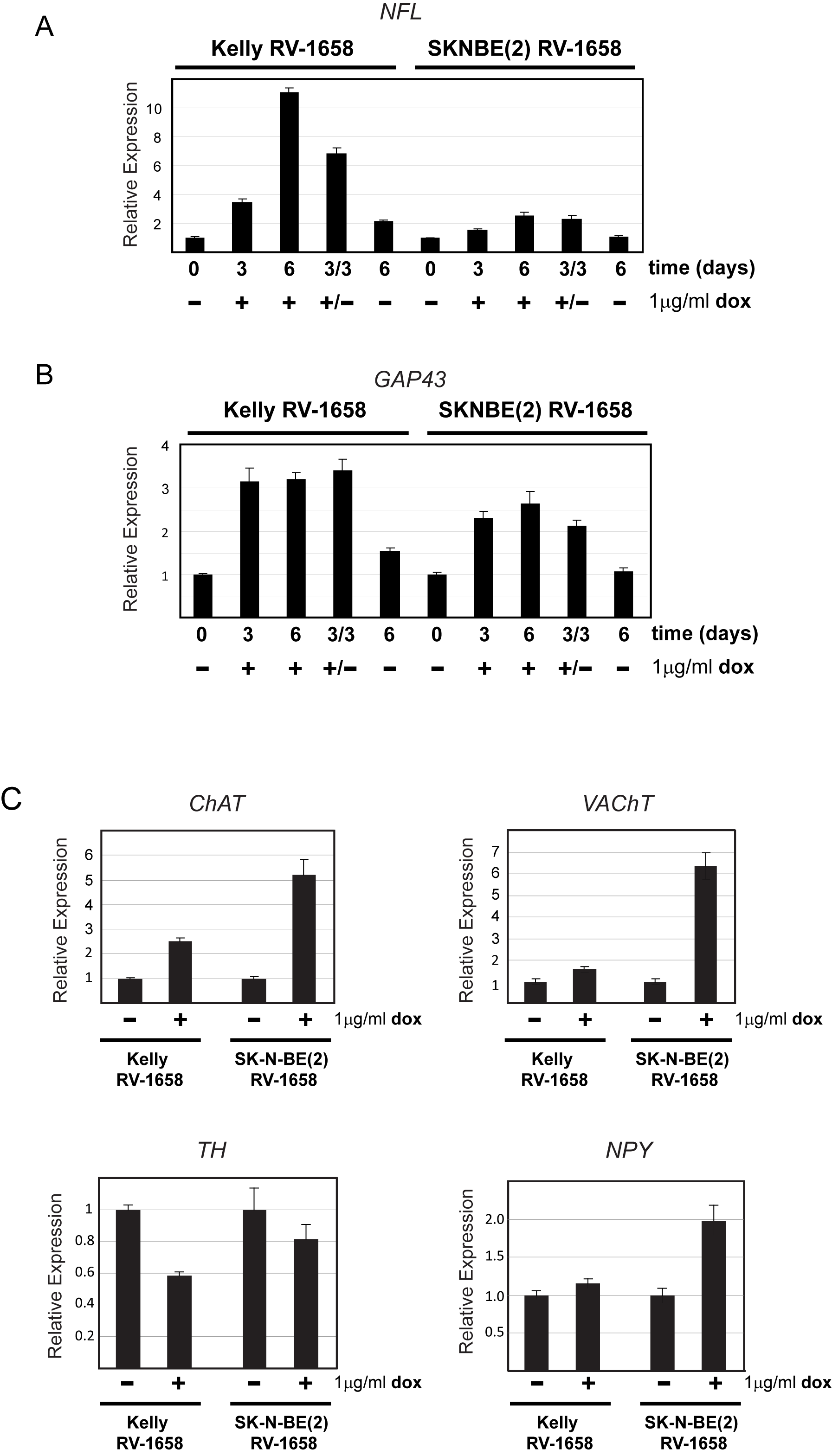

Supplement: Additional file 2 — mRNA expression of neuronal markers in neuroblastoma cell lines after transduction with inducible anti-MYCN shRNA expressing retroviruses. Real-time RT-PCR analysis of NFL (A) and GAP43 (B) mRNA expression in Kelly and SK-N-BE(2) cells transduced with the retrovirus RV-1658. Cells were incubated for the indicated numbers of days in the presence (+) or absence (-) of 1 μg/ml doxycyclin (dox). 3/3 and +/- indicate that the cells were incubated for 3 days in the presence of dox, followed by 3 days in the absence of dox. (C): Real-time RT-PCR analysis of ChAT, VAChT, TH and NPY mRNA in RV-1658 transduced Kelly and SK-N-BE(2) cells grown in the absence (-) or presence (+) of doxycyclin (dox) for 6 days. [file 1471-213X-11-1-S2.PNG]

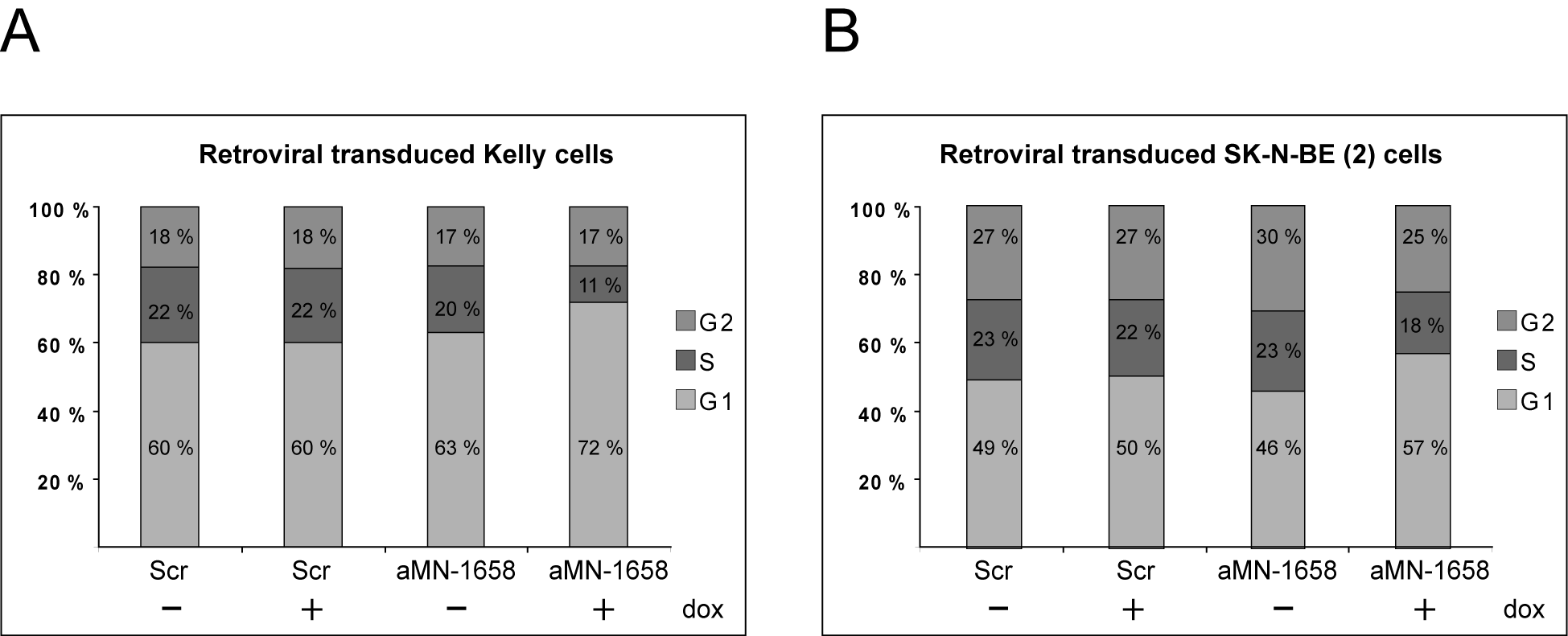

Supplement: Additional file 3 — Cell cycle distribution of neuroblastoma cell lines after transduction with inducible anti-MYCN shRNA expressing retroviruses. Flow cytometric analysis showing the cell cycle distribution of Kelly (A) and SK-N-BE(2) (B) cells transduced with the RV-1658 and RV-Scr retroviruses in the presence (+) or absence (-) of 1 μg/ml doxycyclin (dox). [file 1471-213X-11-1-S3.PNG]
