# Supplementary material for: Conditional expression of retrovirally delivered anti-MYCN shRNA as an in vitro model system to study neuronal differentiation in MYCN-amplified neuroblastoma
Source: BMC Dev Biol. 2011 Jan 3;11:1. doi: 10.1186/1471-213X-11-1 (PMC3022612; doi:10.1186/1471-213X-11-1)
Supplement: Additional file 4 — Oligonucleotides. Oligonucleotides used in this study. [file 1471-213X-11-1-S4.DOC]

| Name | Sequence (5’-3’) |
| --- | --- |
| ON056 | ATTTGGGTCGCGGTTCTTG |
| ON057 | TGCCTTGACATTCTCGATGGT |
| ON058 | GCAGCTACTCCTCCAGCTCT |
| ON059 | ACTTGAGGTCGTTGCTGATG |
| ON060 | TCCAGCCCAGAGACACTGATT |
| ON061 | AGGGTCTTCAAGCCGAGTTCT |
| ON100 | AGATCCCGGAGTTGGAAAAC |
| ON101 | AGCTTTTGCTCCTCTGCTTG |
| ON106 | GATCCCGTTCTTGGGACGCACAGTGATGGTGAATGCAAGCTTCCATTTACCATCGCTGTGTGTCTCAAGAACTTTTTTGGAAA |
| ON107 | AGCTTTTCCAAAAAAGTTCTTGAGACACACAGCGATGGTAAATGGAAGCTTGCATTCACCATCACTGTGCGTCCCAAGAACGG |
| ON110 | GATCCCGAGCGTTCGGAGTTGGTGGTCATAAGTACCAAGCTTCGTATTTATGGCCATCAGCTCCGAACGCTCTTTTTTGGAAA |
| ON111 | AGCTTTTCCAAAAAAGAGCGTTCGGAGCTGATGGCCATAAATACGAAGCTTGGTACTTATGACCACCAACTCCGAACGCTCGG |
| ON145 | GGTCACGGAGATGCTGCTTGAGAAC |
| ON146 | AGAAGCCGCTCCACATGCAGTCC |
| ON174 | GTCAACCCCACCGTGTTCTT |
| ON175 | CTGCTGTCTTTGGGACCTTGT |
| ON298 | ACGACCAAAAGATTGAACAAGATG |
| ON299 | TCCACGGAAGCTAGCCTGAA |
| ON413 | GATCCCGCTGTTGAAGTCACCTTGTGTGTTTCAAGAGAACACACAAGGTGACTTCAACAGTTTTTTGGAAA |
| ON414 | AGCTTTTCCAAAAAACTGTTGAAGTCACCTTGTGTGTTCTCTTGAAACACACAAGGTGACTTCAACAGCGG |
| ON440 | CTAAACGTTGGTGACGGTTG |
| ON441 | GTATCAAATGGCAAACCCCT |
| ON453 | GCACCTTCGCGCAGTTCT |
| ON454 | ACAGCGTGGACAGCTTCTCA |
| ON516 | CCCGGGCCATGTGCAAGGAG |
| ON517 | CCGTGGTTGTGGGCACCTGG |
| ON518 | CCTTCGGCCCCGCTACCCTA |
| ON519 | GGCCCGCTCAAGGGGTTCAC |
